# Reliability and Construct Validity of the Japanese Version of the Posture and Postural Ability Scale in Individuals with Cerebral Palsy

**DOI:** 10.1298/ptr.E10287

**Published:** 2024-05-29

**Authors:** Yuki KIMURA, Yasuaki KUSUMOTO, Hiroto HAYASHI, Natsui KYUJI, Akiho NASU, Hirotaka GIMA

**Affiliations:** ^1^Department of Physical Therapy, Graduate School of Human Health Sciences, Tokyo Metropolitan University, Japan; ^2^Department of Rehabilitation, Sagamihara Ryoikuen, Japan; ^3^Department of Physical Therapy, Fukushima Medical University School of Health Sciences, Japan; ^4^Department of Rehabilitation, Machida Hilltop Hospital, Japan; ^5^Clinical Support HUB, Sunrise Medical Australia, Australia; ^6^Department of Rehabilitation, Nishinomiya Sunago Medical and Welfare Center for Children with Severe Motor and Intellectual Disabilities, Japan; ^7^Department of Rehabilitation, Beppu Developmental Medicine and Rehabilitation Center, Japan

**Keywords:** Cerebral palsy, Posture, Posture and Postural Ability Scale, Reliability, Validity

## Abstract

Objective: This study aimed to develop the Japanese version of the Posture and Postural Ability Scale (PPAS) and verify its inter- and intra-rater reliability, construct validity, and internal consistency in individuals with cerebral palsy (CP) in Japan. Methods: This cross-sectional study recruited 73 children and adults with CP at all Gross Motor Function Classification System (GMFCS) levels. The translation procedure was performed by three Japanese physiotherapists and the developer of the original version. Intra- and inter-rater reliability were evaluated using the weighted kappa coefficients, and construct validity was based on the correlation coefficients between PPAS and GMFCS. Cronbach’s alpha coefficients were calculated to assess internal consistency. Results: Weighted kappa coefficients for intra- and inter-rater reliability exceeded 0.81 for all items. The correlation coefficients between the PPAS and GMFCS were negative and showed “moderate” to “very strong” in almost all items (ρ = −0.66 to −0.91), except for one item (ρ = −0.37). Cronbach’s alpha coefficients exceeded 0.80 in all four positions. Conclusion: This study supports the Japanese version of the PPAS with excellent intra- and inter-rater reliability, good construct validity, and internal consistency in the Japanese CP population.

## Introduction

Cerebral palsy (CP) is defined as “a group of permanent disorders of the development of movement and posture, causing activity limitation, that are attributed to nonprogressive disturbances that occurred in the developing fetal or infant brain. The motor disorders of cerebral palsy are often accompanied by disturbances of sensation, perception, cognition, communication, and behavior, by epilepsy, and by secondary musculoskeletal problems”^1)^, and it is well known that contractures and deformities are among the most common secondary complications in individuals with CP^1^^–^^4)^. Especially, severe individuals classified as level IV or V according to the Gross Motor Function Classification System (GMFCS)^5)^, which classifies gross motor function into five groups, often encounter complicated conditions called “postural deformity”^4^^,^^6^^–^^9)^. Postural deformity includes spinal scoliosis, pelvic obliquity, hip dislocation, and windswept hip deformity^6)^. At birth, there are no contractures or deformities; however, postural deformity is thought to develop along with their growth due to gravity and spending an extended period of time in asymmetric posture^3^^,^^6^^–^^12)^. Postural deformity affects physical appearance, pain increase^13^^–^^15)^, limitations in activities and participation^16)^, and reductions in respiratory function and quality of life^14^^,^^17)^. Therefore, therapists have to assess posture properly and manage these conditions from a preventive perspective at an early stage in life because they do not exist in early infancy^8^^,^^9^^,^^11^^,^^12^^,^^18^^–^^21)^. However, the lack of postural assessment tools for CP in Japan makes its management difficult. In Europe, the Posture and Postural Ability Scale (PPAS) was developed to assess posture in individuals with severe physical disabilities. In the period 1970s–1990s, several postural assessment scales were developed. However, their psychometric properties have not been evaluated^22)^. The PPAS was developed based on these postural assessment scales and showed high psychometric properties such as inter-rater reliability, construct validity, and internal consistency in children and adults with CP^22^^,^^23)^. This scale is reliable and valid, and can identify postural asymmetries at all GMFCS levels in children and adults with CP^22^^,^^23)^. Some studies indicated that national surveillance programs for individuals with CP, including postural assessment, can reduce the incidence of postural deformities such as windswept hip deformity and hip dislocation^3^^,^^24)^.

Although two previous studies have reported high reliability, including inter-rater reliability and internal consistency, and good construct validity of the PPAS^22^^,^^23)^, the psychometric property for intra-rater reliability based on repetitive measurements is still unclear. Additionally, these previous studies involved the developer of the original version of the PPAS, which may have affected the excellent results. Psychometric properties should be examined in different measurement settings. Moreover, a psychometric evaluation study of the Japanese population should also be performed using PPAS in Japan.

The aims of this study were, therefore, to develop the Japanese version of the PPAS and to examine intra- and inter-rater reliability, construct validity, and internal consistency of the Japanese version in individuals with CP in Japan.

## Methods

### Translation procedure

The Japanese version of the PPAS was developed by two physiotherapists who performed forward translation (the primary language was Japanese and had experience in the field of pediatric rehabilitation for several years), one physiotherapist who performed back translation (the primary language was Japanese and had more than 10 years of experience living abroad, and also had back translation experience in the development of Japanese versions of several scales), and Dr. Elisabet Rodby-Bousquet, the University of Lund, the developer of the original version.

First, consent for translation was obtained from the developer. Second, the two translators performed the forward translation based on the original version. Third, we integrated the two versions of the PPAS in Japanese into one, and then one translator performed the back translation. We then confirmed the content of the translation and obtained revisions and additional information from the developer once. Subsequently, we revised the content of the Japanese version based on the revisions and additional information. Finally, we obtained approval for the translation from the developer, and the translation of the PPAS was completed after reviewing typographical errors in the Japanese version.

### Psychometric evaluation of the Japanese version of the PPAS

#### 1) Participants

In this cross-sectional study, participants were recruited from three pediatric rehabilitation facilities in Japan between October 2022 and April 2023. The inclusion criteria were individuals diagnosed with CP. Individuals who underwent orthopedic surgery or botulinum toxin therapy within 6 months were excluded from the study. This study was approved by the Research Ethics Committee of Tokyo Metropolitan University Arakawa Campus (Approval No. 22037). All participants and their families or proxies were fully informed of the outline of the study both orally and in writing, and informed consent was obtained before participation.

#### 2) Measurement

The PPAS assesses “postural ability” (capacity of the movement) and “quality of posture” (positional relationship between the head, trunk, pelvis, extremities, weight distribution, etc.) in four basic positions: supine and prone lying, sitting, and standing. Postural ability is a 7-point ordinal scale and is scored by observing the participant’s possible movement in each position. The examiner rated level 1 (unplaceable) to 7 (moving into and out of position). Quality of posture ranges from 0 to 6 points. It is scored by observing the positional relationships between the head, trunk, pelvis, legs, arms, and weight distribution in the frontal and sagittal planes. For each item, symmetry and midline or neutral alignment scored 1 point, whereas asymmetry and deviation from the midline or neutral position scored 0 points. To assess the quality of posture, examiners instructed or provided manual support to the participants to place them as straight as possible in each position. When a person cannot be placed in a position due to severe contractures or deformities, the postural ability was scored level 1 and automatically scored 0 points for the quality of posture. For the measurements in this study, the first author made a scoring manual of the PPAS, and the developer of the original version approved the content. Co-researchers shared the scoring manual and completed a few hours of training with the first author by using the manual. After pilot testing with a few cases, the first author confirmed the detailed scoring criteria with the developer when it was unclear and not described, and the first author shared information with the co-researchers. The Japanese version of the PPAS and the scoring manual are available freely from supplementary material (referring to Appendix).

The GMFCS is used to classify individuals with CP into one of the five groups based on their gross motor function (levels I–V)^5)^. Higher levels (IV and V) indicate severe motor and functional limitations. In this study, the expanded and revised version of the GMFCS was used, and physiotherapists at each facility determined the gross motor function levels according to their ability to move in daily settings.

#### 3) Data collection and procedures

For intra-rater reliability, one physiotherapist who engaged in the translation procedure of the PPAS (with 3 years of experience in pediatric physical therapy) scored twice within one month by direct observation. All measurements for intra-rater reliability were performed at the same place and setting. Photos and videos were used for the measurements of inter-rater reliability based on a previous study^22)^. When a physiotherapist scored the PPAS for intra-rater reliability, photos and videos of the participant’s movement and posture were taken using an iPad (Apple, Cupertino, CA, USA). Two physiotherapists (13 and 23 years of experience in pediatric physical therapy, respectively) who were not involved in the translation procedure scored once from photos and videos. For construct validity, the GMFCS was rated by physiotherapists at each facility. Data from direct observation measurements of all participants were used to analyze the relationship between PPAS and GMFCS. Additionally, the internal consistency of the scale was evaluated using all the PPAS data.

Physiotherapists at each facility also classified the CP subtype, the Manual Ability Classification System (MACS)^25^^,^^26)^ and the Communication Function Classification System (CFCS)^26^^,^^27)^ to describe the characteristics of participants. CP subtype, MACS, and CFCS were classified based on physical examination and their condition in their daily life.

### Data analysis

In this study, all the PPAS scores were treated as an ordinal scale. Weighted kappa coefficients were calculated for intra- and inter-rater reliability. The quadratic weighted kappa was used in this study. For construct validity, especially for hypotheses testing^28)^, Spearman’s rank correlation coefficient was used to describe the relationship between the PPAS and GMFCS. We hypothesized that lower gross motor function results in lower postural ability and stronger postural asymmetries. The Cronbach’s alpha coefficient was calculated to evaluate the internal consistency of the Japanese version. As the PPAS assesses posture and the ability to move in each position, we assumed high homogeneity among the items. Data were analyzed using the SPSS statistical package for Windows, version 27.0 (IBM, Armonk, NY, USA). The significance level was set at p <0.05.

## Results

### Characteristics of participants

This study included 73 children and adults with CP, with a mean (SD) age of 24.1 (14.5) years. The number of participants who participated in the intra- and inter-rater reliability measurements was 26 and 30, respectively. Table 1 details the participant characteristics: sex, age, height, weight, CP subtype, GMFCS, MACS, and CFCS levels. Median value, interquartile range (IQR), and minimum and maximum values were calculated at each GMFCS level (referring to Appendix).

**Table 1.** The characteristics of participants

	All participants (n = 73)	Intra-rater reliability (n = 26)	Inter-rater reliability (n = 30)
Sex (male, female), n	37, 36	12, 14	13, 17
Age, years	24.0 (14.5) [1–59]	19.0 (16.7) [3–53]	18.1 (9.7) [3–39]
Height, cm	143.3 (19.0)	137.6 (16.7)	138.2 (16.1)
Weight, kg	41.1 (17.9)	31.4 (10.4)	33.2 (11.7)
CP subtype (SBCP, SUCP, Dyskinetic, Ataxic, Mixed, Unclassified), n	60, 2, 3, 2, 5, 1	19, 1, 2, 0, 3, 1	22, 1, 2, 0, 4, 1
GMFCS (I, II, III, IV, V), n	10, 16, 11, 19, 17	4, 4, 1, 7, 10	4, 5, 2, 8, 11
MACS (I, II, III, IV, V), n	22, 12, 14, 7, 18	7, 2, 2, 4, 11	8, 2, 3, 5, 12
CFCS (I, II, III, IV, V), n	29, 12, 13, 4, 15	6, 1, 6, 2, 11	8, 1, 7, 3, 11

Mean (SD: standard deviation) [min–max], CP, cerebral palsy; SBCP, spastic bilateral cerebral palsy; SUCP, spastic unilateral cerebral palsy; GMFCS, Gross Motor Function Classification System; MACS, Manual Ability Classification System; CFCS, Communication Function Classification System

### Intra-rater reliability

Table 2 shows weighted kappa coefficients for intra- and inter-rater reliability. Weighted kappa coefficients for intra-rater reliability ranged from 0.99 to 1.00 in postural ability, 0.93 to 0.98 in quality of posture in the frontal plane, and 0.88 to 0.97 in quality of posture in the sagittal plane.

**Table 2.** Weighted kappa coefficients for intra- and inter-rater reliability

	Intra-rater reliability (n = 26)			Inter-rater reliability (n = 30)		
	Postural ability	Quality of posture in frontal	Quality of posture in sagittal	Postural ability	Quality of posture in frontal	Quality of posture in sagittal
Supine	0.99 (0.98–1.00)	0.96 (0.92–1.00)	0.95 (0.89–1.00)	0.96 (0.91–1.00)	0.81 (0.68–0.95)	0.86 (0.77–0.95)
Prone	0.99 (0.97–1.00)	0.93 (0.84–1.00)	0.97 (0.93–1.00)	0.96 (0.90–1.00)	0.93 (0.86–0.99)	0.89 (0.82–0.97)
Sitting	0.99 (0.97–1.00)	0.94 (0.87–1.00)	0.88 (0.77–0.99)	0.98 (0.95–1.00)	0.92 (0.86–0.98)	0.92 (0.86–0.99)
Standing	1.00 (1.00–1.00)	0.98 (0.96–1.00)	0.95 (0.91–0.99)	1.00 (1.00–1.00)	0.89 (0.79–1.00)	0.82 (0.67–0.97)

Weighted kappa coefficient (95% confidence interval)

### Inter-rater reliability

In this study, two methods were used to measure PPAS: direct observation and observation from photos and videos. Therefore, the agreement between the two types of observations was evaluated using the weighted kappa coefficient. The agreement between the scores from direct observation by one physiotherapist who engaged in the translation procedure and the scores from indirect observation by two physiotherapists who were not involved in the translation procedure and using photos and videos was good because kappa coefficients exceeded 0.75 for all items, respectively.

Weighted kappa coefficients for inter-rater reliability ranged from 0.96 to 1.00 in postural ability, 0.81 to 0.93 in quality of posture in the frontal plane, and 0.82 to 0.92 in quality of posture in the sagittal plane (Table 2).

### Hypotheses testing for construct validity

**Fig. 1.**
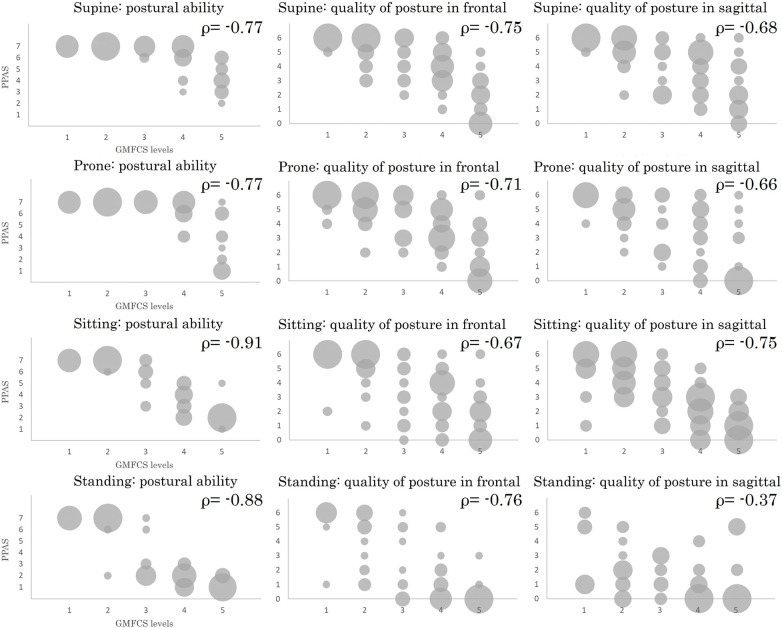
Distribution of PPAS scores at each GMFCS level This figure shows the distribution of the PPAS scores at each GMFCS level with a bubble chart. The size of each circle represents the percentage of a number in all participants (n = 73) ρ, Spearman’s rank correlation coefficient; PPAS, Posture and Postural Ability Scale; GMFCS, Gross Motor Function Classification System

Figure 1 shows the distribution of PPAS and GMFCS levels for each item using a bubble chart. Correlation coefficients between the PPAS and GMFCS were −0.77 to −0.91 in postural ability, −0.67 to −0.76 in quality of posture in the frontal plane, −0.37 to −0.75 in quality of posture in the sagittal plane (Table 3). In addition, the correlation between PPAS scores and age was analyzed using Spearman’s rank correlation coefficient, and all items showed low values (Table 3).

**Table 3.** Correlation coefficients between PPAS and GMFCS, age

	GMFCS	Age
Supine		
Postural ability	−0.77^**^ (−0.66 to −0.85)	0.05 (−0.19 to 0.27)
Quality of posture in frontal	−0.75^**^ (−0.63 to −0.84)	−0.19 (−0.40 to 0.04)
Quality of posture in sagittal	−0.68^**^ (−0.53 to −0.79)	−0.26^*^ (−0.03 to −0.46)
Prone		
Postural ability	−0.77^**^ (−0.65 to −0.85)	0.03 (−0.20 to 0.26)
Quality of posture in frontal	−0.71^**^ (−0.57 to −0.81)	−0.23 (−0.43 to 0.00)
Quality of posture in sagittal	−0.66^**^ (−0.51 to −0.78)	−0.17 (−0.38 to 0.06)
Sitting		
Postural ability	−0.91^**^(−0.86 to −0.94)	−0.06 (−0.28 to 0.17)
Quality of posture in frontal	−0.67^**^ (−0.52 to −0.78)	−0.22 (−0.43 to 0.01)
Quality of posture in sagittal	−0.75^**^ (−0.62 to −0.83)	−0.06 (−0.29 to 0.17)
Standing		
Postural ability	−0.88^**^ (−0.81 to −0.92)	−0.07 (−0.29 to 0.16)
Quality of posture in frontal	−0.76^**^ (−0.64 to −0.84)	−0.18 (−0.39 to 0.05)
Quality of posture in sagittal	−0.37^**^ (−0.15 to −0.55)	−0.25^*^ (−0.02 to −0.46)

Correlation coefficient (95% confidence interval)

^^**^^p < 0.01, *p < 0.05

ρ, Spearman’s rank correlation coefficient; n = 73

GMFCS, Gross Motor Function Classification System

### Internal consistency

Table 4 shows Cronbach’s alpha coefficients for internal consistency in each position. Cronbach’s alpha coefficients were supine: 0.87, prone: 0.90, sitting: 0.88, and standing: 0.85.

**Table 4.** Cronbach’s alpha coefficient in each position

	Cronbach’s alpha	Cronbach’s alpha if item deleted	Corrected item – total correlation
Supine			
Total	0.87	–	–
Postural ability	–	0.93	0.64
Quality of posture in frontal	–	0.70	0.88
Quality of posture in sagittal	–	0.78	0.81
Prone			
Total	0.90	–	–
Postural ability	–	0.92	0.72
Quality of posture in frontal	–	0.78	0.89
Quality of posture in sagittal	–	0.86	0.80
Sitting			
Total	0.88	–	–
Postural ability	–	0.88	0.72
Quality of posture in frontal	–	0.86	0.74
Quality of posture in sagittal	–	0.76	0.85
Standing			
Total	0.85	–	–
Postural ability	–	0.78	0.75
Quality of posture in frontal	–	0.69	0.83
Quality of posture in sagittal	–	0.87	0.67

## Discussion

### Reliability

For intra- and inter-rater reliability, kappa coefficients exceeded 0.81 for all items in this study. The kappa coefficient ranges from 0 to 1 and is generally considered a moderate agreement between 0.41 and 0.60, a substantial agreement between 0.61 and 0.80, and an almost perfect agreement at 0.81 and higher^29)^. Therefore, the results indicated that the Japanese version of the PPAS has excellent intra- and inter-rater reliability.

Kappa coefficients for inter-rater reliability based on the agreement between two raters in this study were slightly lower than those of a previous study, in which measurements were performed by three raters who developed the original version of the PPAS^22)^. However, the results were similar to those of another study performed by three raters, where two were not involved in the development of the original version^23)^.

In this study, the PPAS was measured by one rater who was engaged in developing the Japanese version and by raters who were not involved in developing the Japanese version but carefully checked the scoring criteria in advance. Therefore, it is possible that different results would be obtained if the PPAS were measured by raters who were not involved in the development of the Japanese version or in the measurement of the PPAS in this study. In the future, when the Japanese version of the PPAS is used in clinical practice, raters should confirm the detailed scoring criteria in advance.

### Construct validity

The correlation coefficients between PPAS and GMFCS were negative for all items. This result supports our hypothesis that lower gross motor function results in lower postural ability and stronger postural asymmetries. The correlation coefficient is considered negligible when it is less than 0.10, weak at 0.10 to 0.39, moderate at 0.40 to 0.69, strong at 0.70 to 0.89, and very strong at 0.90 or higher^30)^. In this study, a weak correlation was confirmed for the item of quality of posture in the sagittal plane in the standing position (ρ = −0.37), and moderate to very strong correlations were confirmed for all other items (ρ = −0.66 to −0.91), indicating that the Japanese version of the PPAS has good construct validity in individuals with CP.

The reason for the relatively low correlation coefficient between the quality of posture in the sagittal plane in a standing position and GMFCS was that, first, the score in the sagittal plane in a standing position tended to be low in those who were equivalent to GMFCS level IV or V because they had difficulty assuming standing posture or had contractures of the trunk or lower extremities. In addition, some individuals equivalent to GMFCS level I or II, who could walk and run, also had low scores for this item. Therefore, the correlation coefficient for this item was low. Some individuals with CP who can walk show lumbar hyperlordosis in standing and crouched standing posture with flexion of the hip and knee joints^31^^–^^34)^, which may have influenced the score in standing position. Since the standing posture mentioned above may lead to secondary symptoms such as low back pain^32)^ and knee pain during gait^35)^, the PPAS may be useful for individuals with severe CP, and those with CP who can walk and run.

Additionally, the correlation coefficients between the PPAS and age ranged from negligible (ρ <0.10) to weak (ρ = 0.10−0.39) for all items, suggesting that age has little association with postural ability and postural asymmetries. In the present study, we did not set the inclusion criterion for age because a previous study suggested that postural asymmetries can be identified in younger children before the age of 3 years^21)^. Abnormalities in muscle tone and/or muscle imbalance^6^^,^^36)^ and early development of joint contractures in knee or ankle joints in early childhood^10)^ may have affected the results of correlations between PPAS and age. Therefore, it is desirable to assess posture from early childhood, when postural asymmetry is recognized but deformity has not developed, and follow up from a preventive perspective of contractures and deformities.

### Internal consistency

The Cronbach’s alpha coefficient is generally considered an indicator of internal consistency, and although there is no absolute standard, a value of 0.80 or higher is required^37)^. In the present study, Cronbach’s alpha coefficients were all greater than 0.80 in all four positions, which supports the good internal consistency of the Japanese version of the PPAS. Additionally, the corrected item-total correlation was used to explain the coherence between an item and other items^38)^; correlations seemed to be good for each item at all four positions.

### Limitations of this study

This study has several limitations. First, although the posture of individuals with severe disabilities may change during the day due to fatigue and pain^22)^, measurements for intra-rater reliability in this study were performed on different days within one month because body functional and structural changes were assumed to be little within one month. Therefore, there is a possibility that differences in the observed postures occurred due to the participant’s physical condition, including fatigue and pain, on each day when the measurements for intra-rater reliability were performed. Furthermore, measurements were performed within a short period of approximately 10 min per session. The examiners might not be able to observe the participant’s possible performance, which they can usually perform in their usual daily life situations, such as at home or school when verbal instructions are difficult owing to their cognitive function. Finally, the sample sizes in the present study for internal consistency and hypotheses testing for construct validity were adequate; however, the sample sizes for intra- and inter-rater reliability were small, respectively, based on the 4-point scale in the COSMIN study design checklist^39)^.

In future research, the criterion validity and therapeutic effects, such as botulinum toxin therapy, orthopedic surgery, and postural changes due to the use of welfare devices, should be investigated longitudinally in Japan.

## Conclusion

This study supports the Japanese version of the PPAS with excellent intra- and inter-rater reliability, good construct validity and internal consistency. This scale can be applied to children and adults in individuals with CP and enables Japanese clinicians to assess posture and postural asymmetries at all GMFCS levels.

## Acknowledgments

The authors would like to thank Dr. Elisabet Rodby-Bousquet for sharing her knowledge, and all children and adults who participated in the study, their families, and therapists, Shigeru Higuchi, Hiroaki Fukasawa, and Yuki Ishida for data collection.

## Funding

This study was supported by France Bed Medical Home Care Research Subsidy Public Interest Incorporated Foundation.

## Conflicts of Interest

The authors declare no conflicts of interest.

## Supplementary Material

Supplementary Material 1 Scoring manual Japanese version of the PPAS

Supplementary Material 2 Japanese version of the PPAS

Supplementary Material 3 Appendix score distribution
