# Supplementary material for: Reliability and Construct Validity of the Japanese Version of the Posture and Postural Ability Scale in Individuals with Cerebral Palsy
Source: Phys Ther Res. 2024 May 29;27(2):92–9. doi: 10.1298/ptr.E10287 (PMC11382793; doi:10.1298/ptr.E10287)
Supplement: Supplementary Material 1 — Scoring manual Japanese version of the PPAS [file ptr-27-92-s01.pdf]

Posture and Postural Ability Scale　スコアリングマニュアル

●Posture and Postural Ability Scale（PPAS）は重度障がい児者（脳性麻痺など）の姿勢と姿勢能力を評価するものです。

●非対称な姿勢は時間経過とともに脊柱側弯や股関節脱臼、風に吹かれた股関節変形などにつながることが考えられます。非対称な姿勢を評価し、早期発見・早期治療につなげることで重度の変形・拘縮を予防していくことが望まれます。本評価ツールはその一助となることが期待されます。

●海外では脳性麻痺患者において信頼性と妥当性が報告されています（2022 年 8 月現在)。

採点について

●PPAS は背臥位・腹臥位・座位・立位の 4 姿勢を評価します。

●口頭指示が可能な場合「できるだけ真っすぐに」のように指示をします。口頭指示が困難な場合、出来るだけ真っすぐな姿勢をとった上で、その後落ち着く姿勢（習慣的な姿勢）を評価の対象とします。

●自力で姿勢をとることができない場合、介助者が各姿勢への姿勢変換を補助し、必要に応じて徒手的な介助を実施します。

1. まず、各姿勢において対象者の可能な運動（姿勢能力）を評価し、レベル 1～7 で採点します。
2. 次に、各姿勢のアライメントを評価します。前額面・矢状面それぞれ 6 点満点で採点します。

※重度の拘縮等により各姿勢をとることが困難な場合はレベル 1、前額面/矢状面は自動的に各 0 点となります。

※座位の評価では、座面の高さにより足部が浮く可能性があるため、必要に応じて座面の高さを調整する、足部の高さを調整する等の工夫が必要な場合があります。

【参考文献】

Rodby-Bousquet, E., Agústsson, A., Jónsdóttir, G., Czuba, T., Johansson, A. C., & Hägglund, G. (2014). Interrater reliability and construct validity of the Posture and Postural Ability Scale in adults with cerebral palsy in supine, prone, sitting and standing positions. Clinical rehabilitation, 28(1),82-90. 他

【日本語版作成者】

林寛人、久司夏井、木村優希、楠本泰士、儀間裕貴

【日本語版お問い合わせ】

木村優希（東京都立大学大学院）　kimurayki326@gmail.com

林寛人（Sunrise Medical Australia）　hiroto.hayashi@sunrisemedical.com.au

2022 年 8 月作成
