# Supplementary material for: Reliability and Construct Validity of the Japanese Version of the Posture and Postural Ability Scale in Individuals with Cerebral Palsy
Source: Phys Ther Res. 2024 May 29;27(2):92–9. doi: 10.1298/ptr.E10287 (PMC11382793; doi:10.1298/ptr.E10287)
Supplement: Supplementary Material 2 — Japanese version of the PPAS [file ptr-27-92-s02.pdf]

Posture and Postural Ability Scale,　　氏名 ________________　日付 ________

背臥位における姿勢能力（Postural ability）：量的評価

（マットやベッド上にておこなう）		
レベル1	背臥位をとることができない（例：重度脊柱後弯）	
レベル2	他動的に背臥位をとることは可能だが、介助が必要	
レベル3	他動的に背臥位をとることで、<br>背臥位の保持は可能だが、動くことは困難	
レベル4	体幹をわずかでも屈曲することができる<br>（頭部や膝を挙上するために体幹を安定させることができる）	
レベル5	側方へ体重移動し、再び背臥位に戻ることができる<br>（側方に寝返りができる）	
レベル6	背臥位から姿勢を変えることができる<br>（腹臥位に寝返りすることができる）	
レベル7	背臥位から、または背臥位へ自由に姿勢を変えることができる<br>（座位になり、再び背臥位をとる）	

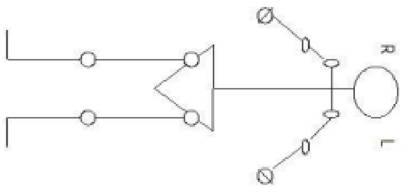

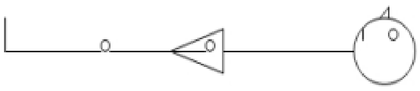

背臥位における姿勢の質（Quality of posture）：質的評価

**前額面（スコア 1=yes, 0=no）**	
頭部は正中位である	
体幹は左右対称である	
骨盤は中間位である	
両下肢は互いに接触せず、<br>骨盤に対して垂直である	
上肢は安楽位で体側に位置している（何かにつかまったり、挙上位でないこと）	
体重分布は均等である	
**合計点**	

**矢状面（スコア 1=yes, 0=no）**	
頭部は正中位（平らな枕使用時）	
体幹は中間位である	
骨盤は中間位である	
下肢は真っすぐであり、<br>股・膝関節は伸展位である	
足部は安楽な肢位である	
体重分布は均等である<br>（肩甲帯から骨盤にかけて）	
**合計点**	

Posture and Postural Ability Scale,　氏名 ________________　日付 ________

腹臥位における姿勢能力（Postural ability）：量的評価

（マットやベッド上にておこなう）		
レベル1	腹臥位をとることができない（例：股関節屈曲拘縮）	
レベル2	他動的に腹臥位をとることは可能だが、介助が必要	
レベル3	他動的に腹臥位をとることで、<br>腹臥位の保持は可能だが、動くことは困難	
レベル4	体幹をわずかでも伸展することができる<br>（頭部を挙上したり、自由に動かす）	
レベル5	側方へ体重移動し、再び腹臥位に戻ることができる<br>（側方に寝返りができる）	
レベル6	腹臥位から姿勢を変えることができる<br>（背臥位に寝返りすることができる）	
レベル7	腹臥位から、または腹臥位へ自由に姿勢を変えることができる<br>（ずり這いをし、再び腹臥位に戻る）	

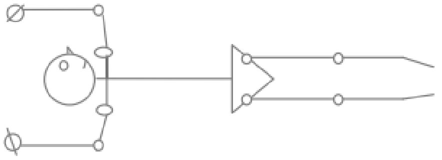

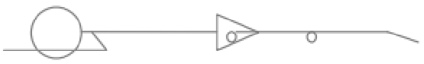

腹臥位における姿勢の質（Quality of posture）：質的評価

前額面（スコア 1=yes, 0=no）	
頭部は一側を向いている	
体幹は左右対称である	
骨盤は中間位である	
両下肢は互いに接触せず、<br>骨盤に対して垂直である	
上肢は安楽位で挙上しており、<br>肘が約90°屈曲位である	
体重分布は均等である	
**合計点**	

矢状面（スコア 1=yes, 0=no）	
体幹は中間位である	
骨盤は中間位である	
股関節は伸展位である<br>（足部は台の端から出した状態）	
膝関節は伸展位である<br>（足部は台の端から出した状態）	
上肢は安楽位で挙上しており、<br>肘が約90°屈曲位である	
体重分布は均等である<br>（肩甲帯から骨盤にかけて）	
**合計点**	

Posture and Postural Ability Scale,　　氏名 ________________　日付 ________

座位における姿勢能力（Postural ability）：量的評価

（足部が接地した状態で台やベッドの端に座る）		
レベル1	座位をとることができない	
レベル2	他動的に座位をとることは可能だが、介助が必要	
レベル3	他動的に座位をとることで、<br>座位の保持は可能だが、動くことは困難	
レベル4	脊柱を曲げることなく、<br>基底面上で体幹をわずかに前後方向に動かすことができる	
レベル5	側方へ体重移動し再び元の姿勢に戻ることができる<br>（一側の臀部から、反対側の臀部に移動する）	
レベル6	座位から姿勢を変えることができる<br>（足部に荷重し、臀部を座面から離す）	
レベル7	座位から、または座位へ自由に姿勢を変えることができる<br>（立ち座りができる）	

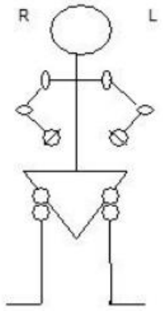

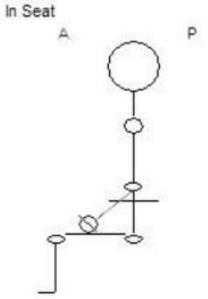

座位における姿勢の質（Quality of posture）：質的評価

**前額面（スコア 1=yes, 0=no）**	
頭部は正中位である	
体幹は左右対称である	
骨盤は中間位である	
両下肢は互いに接触せず、<br>骨盤に対して垂直である	
上肢は安楽位で体側に位置している(何かにつかまったり、挙上位でないこと)	
体重分布は均等である	
**合計点**	

**矢状面（スコア 1=yes, 0=no）**	
頭部は正中位である	
体幹は中間位である	
骨盤は中間位である	
股関節は90°屈曲位である	
膝関節は90°屈曲位である	
足部は中間位で全面で接地している	
**合計点**	

Posture and Postural Ability Scale,　　氏名 ________________　日付 ________

立位における姿勢能力（Postural ability）：量的評価

レベル1	立位をとることができない（例：股関節屈曲拘縮）	
レベル2	他動的に立位をとることは可能だが、介助が必要	
レベル3	他動的に立位をとることで、<br>立位の保持は可能だが、動くことは困難	
レベル4	脊柱を曲げることなく、<br>基底面上で体幹をわずかに前後方向に動かすことができる	
レベル5	側方へ体重移動し再び元の姿勢に戻ることができる<br>（一側の足部に荷重し、反対側へ移動する）	
レベル6	立位から姿勢を変えることができる<br>（前方へステップする）	
レベル7	立位から、または立位へ自由に姿勢を変えることができる<br>（数歩歩行し、立ち止まったりできる）	

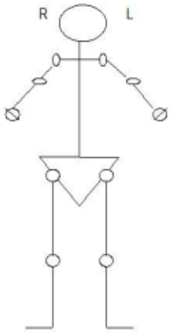

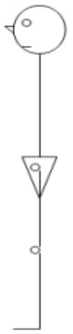

立位における姿勢の質（Quality of posture）：質的評価

前額面（スコア 1=yes, 0=no）	
頭部は正中位である	
体幹は左右対称である	
骨盤は中間位である	
両下肢は互いに接触せず、<br>骨盤に対して垂直である	
上肢は安楽位で体側に位置している（何かにつかまったり、挙上位でないこと）	
体重分布は均等である<br>（両足部において）	
**合計点**	

矢状面（スコア 1=yes, 0=no）	
頭部は正中位である	
体幹は中間位である	
骨盤は中間位である	
下肢は真っすぐで、<br>股・膝関節は伸展位である	
足部は中間位で全面で接地している	
体重分布は均等である<br>（足部において）	
**合計点**	
