# Supplementary material for: Reliability and Construct Validity of the Japanese Version of the Posture and Postural Ability Scale in Individuals with Cerebral Palsy
Source: Phys Ther Res. 2024 May 29;27(2):92–9. doi: 10.1298/ptr.E10287 (PMC11382793; doi:10.1298/ptr.E10287)
Supplement: Supplementary Material 3 — Appendix score distribution [file ptr-27-92-s03.pdf]

Appendix table. Detailed score distribution of the Japanese version of the Posture and Postural Ability Scale

	GMFCS level	I (n=10)	II (n=16)	III (n=11)	IV (n=19)	V (n=17)
		Median [IQR] (Min-Max)	Median [IQR] (Min-Max)	Median [IQR] (Min-Max)	Median [IQR] (Min-Max)	Median [IQR] (Min-Max)
Supine	Postural ability	7.0 [7.0-7.0] (7-7)	7.0 [7.0-7.0] (7-7)	7.0 [7.0-7.0] (6-7)	7.0 [6.0-7.0] (3-7)	4.0 [3.0-5.5] (2-6)
	Quality of posture in frontal	6.0 [6.0-6.0] (5-6)	6.0 [4.3-6.0] (3-6)	5.0 [3.0-6.0] (2-6)	4.0 [3.0-5.0] (1-6)	2.0 [0.0-3.0] (0-5)
	Quality of posture in sagittal	6.0 [6.0-6.0] (5-6)	5.0 [5.0-6.0] (2-6)	4.0 [2.0-5.0] (2-6)	4.0 [2.0-5.0] (1-6)	2.0 [1.0-4.0] (0-6)
Prone	Postural ability	7.0 [7.0-7.0] (7-7)	7.0 [7.0-7.0] (7-7)	7.0 [7.0-7.0] (7-7)	7.0 [6.0-7.0] (4-7)	3.0 [1.0-6.0] (1-7)
	Quality of posture in frontal	6.0 [5.8-6.0] (4-6)	5.0 [5.0-6.0] (2-6)	5.0 [3.0-6.0] (2-6)	3.0 [3.0-5.0] (1-6)	1.0 [0.0-3.0] (0-6)
	Quality of posture in sagittal	6.0 [6.0-6.0] (4-6)	5.0 [4.0-5.8] (2-6)	4.0 [2.0-6.0] (1-6)	3.0 [1.0-5.0] (0-6)	0.0 [0.0-3.0] (0-6)
Sitting	Postural ability	7.0 [7.0-7.0] (7-7)	7.0 [7.0-7.0] (6-7)	6.0 [5.0-7.0] (3-7)	4.0 [2.0-4.0] (2-5)	2.0 [2.0-2.0] (1-5)
	Quality of posture in frontal	6.0 [6.0-6.0] (2-6)	6.0 [5.0-6.0] (1-6)	3.0 [1.0-5.0] (0-6)	4.0 [2.0-4.0] (0-6)	2.0 [0.0-2.5] (0-6)
	Quality of posture in sagittal	5.5 [4.5-6.0] (1-6)	5.0 [4.0-6.0] (3-6)	3.0 [2.0-5.0] (1-6)	2.0 [1.0-3.0] (0-5)	1.0 [0.0-2.0] (0-3)
Standing	Postural ability	7.0 [7.0-7.0] (7-7)	7.0 [7.0-7.0] (2-7)	2.0 [2.0-3.0] (2-7)	2.0 [1.0-2.0] (1-3)	1.0 [1.0-1.5] (1-2)
	Quality of posture in frontal	6.0 [5.8-6.0] (1-6)	5.0 [2.0-6.0] (1-6)	1.0 [0.0-5.0] (0-6)	1.0 [0.0-2.0] (0-5)	0.0 [0.0-0.0] (0-3)
	Quality of posture in sagittal	3.0[1.0-5.3] (1-6)	2.0 [0.3-2.8] (0-5)	2.0 [1.0-3.0] (0-3)	0.0 [0.0-1.0] (0-4)	0.0 [0.0-3.5] (0-5)

IQR: interquartile range.
